# Pain assessment in children undergoing venipuncture: the Wong–Baker faces scale versus skin conductance fluctuations

**DOI:** 10.7717/peerj.37

**Published:** 2013-02-12

**Authors:** Francesco Savino, Liliana Vagliano, Simone Ceratto, Fabio Viviani, Roberto Miniero, Fulvio Ricceri

**Affiliations:** 1 Città della Salute e della Scienza di Torino, Regina Margherita Children’s Hospital, Dipartimento di Scienze della Sanità Pubblica e Pediatriche, University of Turin, Italy; 2 Experimental Pediatrics, Department of Pediatrics, Doctoral School in Biomedical Sciences and Human Oncology, University of Turin, Italy; 3 Department of Internal Medicine and Surgery, Università della Magna Grecia, Catanzaro, Italy; 4 Human Genetics Foundation, Turin, Italy

**Keywords:** Pain assessment, Wong–Baker scale, skin conductance, venipuncture, children

## Abstract

The aim of this study was to evaluate the efficacy of the subjective Wong–Baker faces pain rating scale (WBFS) and of the objective skin conductance fluctuation (SCF) test in assessing pain in children undergoing venipuncture. One-hundred and fifty children (aged 5–16 years) entered the study. All underwent venipuncture at the antecubital fossa to collect blood specimens for routine testing in the same environmental conditions. After venipuncture, the children indicated their pain intensity using the WBFS, whereas the number of SCFs was recorded before, during and after venipuncture. So, pain level was measured in each child with WBFS and SCF. We found that the level of WBFS-assessed pain was lower in all children, particularly those above 8 years of age, than SCF-assessed pain (*p* < 0.0001). Moreover, the number of SCFs was significantly higher during venipuncture than before or after venipuncture (*p* < 0.0001). At multivariate regression analysis, age and previous experience of venipuncture influenced the WBFS (β = −1.81, *p* < 0.001, and β = −0.86, *p* < 0.001, respectively) but not SCFs. In conclusion, although both procedures can be useful for research and clinical practice, our findings show that WBFS was affected by age and previous venipuncture, whereas SCF produced uniform data. If verified in other studies, our results should be taken into account when using these tools to evaluate pain in children.

## Introduction

During the routine care of children, painful invasive procedures such as venipuncture for the withdrawal of blood for hematological testing are usually inevitable in healthy and sick subjects. The importance of pain assessment and pain management is widely acknowledged (Drendel, Kelly & Ali, 2011), and alleviation of pain caused by minor invasive procedures in children is an important issue for humane reasons and in terms of their reactions to future painful events and acceptance of subsequent health care interventions; (von Baeyer et al., 2004) moreover, unrecognized pain can become severe and difficult to control and lead to fear and stress (Roeggen, 2009).

Pain assessment is an ongoing and integral part of total pain management particularly in children, and includes such approaches as distraction, evaluation, reassessment and medical intervention (Davidson & McKenzie, 2011; Eichenfield et al., 2002; Mathew & Mathew, 2003; Taddio et al., 2011). Children and adolescents often describe invasive procedures and their associated anticipatory anxiety as the most distressing aspect of illness or hospitalization (von Baeyer et al., 2004). Venipuncture is one of the most feared and acute painful experience in children (McMurtry et al., 2011).

The main difficulty in assessing pain in children is the potential discrepancy between the perception and experience of pain and its expression (Alexander et al., 1993; Davidson & McKenzie, 2011; Eichenfield et al., 2002; Mathew & Mathew, 2003; Roeggen, 2009; Taddio et al., 2011; von Baeyer et al., 2004). Self-report faces scales are widely used to evaluate pain intensity in children despite concerns regarding interpretability (Stinson et al., 2006; Tomlinson et al., 2010). Most scales have five to seven faces, which are intended to elicit an indication of pain intensity (Beyer & Aradine, 1987; Hicks et al., 2001; Kuttner, 1989; LeBaron & Zeltzer, 1984; Wallin, Sundlof & Delius, 1975). This gives more information than a simple binary “pain”/“no pain” response.

Faces scales are frequently used as self report measures of pain intensity in research and clinical practice, and the Royal College of Nursing has identified the WBFS as suitable for peri-procedural pain (Roeggen, 2009).

Various pain assessment tools have been tested in the search for objective, specific physiologic measures of responses to pain in infants (Stapelkamp et al., 2011). One of the most investigated is skin conductance fluctuations (SCF) per sec measured in the palm of the hand or on the plantar aspect of the foot (Tranel & Damasio, 1994). Skin conductance fluctuations in these sites reflect emotional sweating due to skin sympathetic nerve activity. They occur within 1–2 s of the onset of emotional stressors such as pain (Hagbarth et al., 1972; Wallin, 1978). Because SCF are induced by acetylcholine acting on muscarinic receptors, they are not affected by environmental temperature, hemodynamic changes or respiratory rhythm medications such as beta blockers and neuromuscular blockers (Bini et al., 1980; Macefield & Wallin, 1996; Wallin, 1978). Furthermore, changes in respiratory rhythm (including apnea) do not influence SCF (Habler et al., 1993; Hanada, Sander & Gonzalez-Alonso, 2003; Valkenburg et al., 2012). Thus, changes in skin conductance are considered useful to monitor nociceptive stimulation and pain (Storm, 2008). The test has also been used to identify increased emotional stress as reflected in changes in the sympathetic nervous system as a measure of discomfort in artificially ventilated children (Gjerstad et al., 2008) and in other conditions (Eriksson et al., 2008; Harrison et al., 2006; Hellerud & Storm, 2002; Ledowski et al., 2006; Munsters et al., 2012; Roeggen, Storm & Harrison, 2011; Storm et al., 2002; Storm et al., 2005). However, also such sympathetic nerve activity as skin temperature autoregulation cause skin conductance peaks that are generally observed in pain states (Valkenburg et al., 2012).

Many pain management studies have focused on postoperative and chronic pain (Goddard, 2011; Lewandowski et al., 2010). However, although the simple insertion of a needle is one of the most frightening and distressing medical procedures for hospitalized children (Alexander et al., 1993; von Baeyer et al., 2004) the recognition and assessment of acute pain resulting from this procedure in children remains inadequate (Drendel, Kelly & Ali, 2011). We have evaluated simultaneously the efficacy of the Wong–Baker Faces Pain Rating Scale (WBFS), which is routinely used in our hospital and SCF per sec in measuring pain in children at different ages (with and without previous exposure to venipuncture), before, during and after venipuncture performed at the same anatomic location (antecubital fossa) (Wong & Baker, 1988).

## Methods

### Study design

This was a prospective, observational study designed to explore relationships and differences between the WBFS to and the SCF during venipuncture in children 5–16 years old.

### Patients

This analytical, observational study was undertaken in the Day Hospital of the Pediatric Specialist and Laboratory Analysis Service of the Children Hospital “Regina Margherita” (Turin, Italy). It was conducted between March 2010 and March (inclusive) 2011 and in accordance with good clinical practice and the Declaration of Helsinki. The protocol was approved by the Ethics Committee of the Azienda Ospedaliera, OIRM S. Anna – Ospedale Mauriziano (Turin, Italy).

We know that WBFS and SCF values are hardly to compare, but we decided to use a conversion table provided by the developer of SCF tool which made possible to convert the SCF values in a graded scale from zero to ten similar to WBFS grades.

A convenience sample of pediatric patients (5–16 years old) was enrolled: eligible patients were children or adolescents candidates for venipuncture for the collection of diagnostic blood specimens for routine hematological testing. All the patients were recruited at the “Regina Margherita” Children Hospital: some were recruited in the outpatient clinic and others were enrolled in Day Hospital, where they were followed for previous gastrointestinal diseases, endocrine disorders or diabetes mellitus type I. Children with known cognitive impairments, developmental delays, sensory deficits, pathological conditions of the palm and children receiving analgesic drugs were excluded from the study. Written consent was obtained from the parents, and verbal assent was obtained from each child or adolescent, adequately informed about the purpose of the study and the tools used. Parents were informed that their acceptance or refusal of the study would not affect clinical service. Basic demographic data (age, gender, previous diseases) were recorded. All the eligible children had to be subjected to venipuncture according to a previous clinical prescriptions in order to obtain diagnostic information.

All the venipunctures for the collection of blood were performed in the same setting with the same environmental temperature and by the same person. In our hospital nonpharmacologic distraction techniques are routinely perfomed: for venipuncture all the subjects received parental holding and positioning.

## Pain evaluation tools

### Wong–Baker faces scale

**Figure 1:**
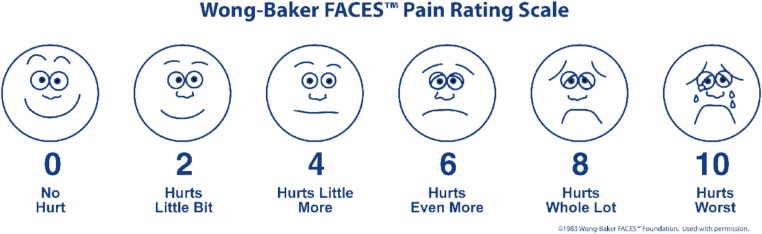
Wong–Baker Faces™ Pain Rating Scale. Reproduced with Permission of the Wong–Baker Faces Foundation™.

The WBFS combines pictures and numbers to enable the user to rate pain (Fig. 1). It can be used for children over the age of 3, and for adults. The faces range from a smiling face to a sad, crying face. A numerical rating is assigned to each face (from 0, “no hurt” to 10, “hurts worst”) of the WBFS (Drendel, Kelly & Ali, 2011; Garra et al., 2010; Hicks et al., 2001; Kuttner, 1989; Stapelkamp et al., 2011; Stinson et al., 2006; Tomlinson et al., 2010).

The WBFS also has adequate psychometric properties (reliability, validity), and it is easy and quick to. The greatest strength of this scale may be its acceptability, given the consistent finding that the WBFS was preferred by children (any age), parents, and practitioners when compared with other faces pain scales (Tomlinson et al., 2010).

Concerning validity, WBFS has an high correlation (*r* > 0.7) with other self-reported pain scale used at the same time and shows differences (*p* < 0.05) in score between two comparable but different groups. Reliability has been proved by the use of “test and retest” (*r* > 0.5) and by the concordance with simultaneous observational score (*r* > 0.4). WBFS has a significant (*p* < 0.05) responsiveness to pain-increasing (painful procedures) and pain-decreasing (analgesia) events (Tomlinson et al., 2010).

### Skin conductance test. Instruments

**Figure 2:**
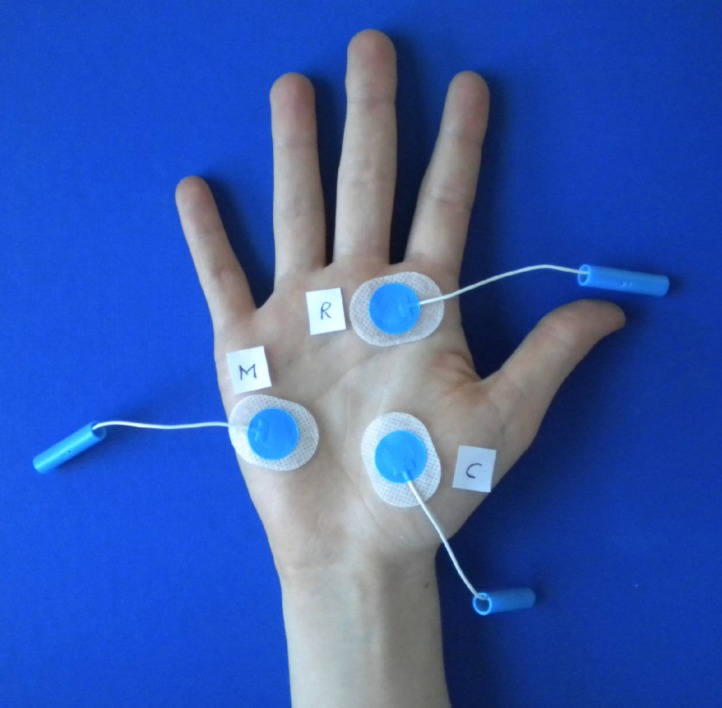
Placement of electrodes.

Skin conductance fluctuations were measured by alternating current at 88 Hz. Low-frequency electrical conductance reflects the ionic conduction in the stratum corneum, which is largely determined by sweat duct filling. Resistance and conductance are two related quantities in measuring voltage changes in skin conductance. Siemens is the unit of electrical conductance in the same way as Ohm is the unit of electrical resistance (Storm, 2008). Conductance was preferred to resistance because of the parallel nature of the electrical polarization and conductance in the skin. A frequency of 88 Hz is sufficient to reduce considerably the requirements for low electrode polarizability but also low enough to ensure minimal influence from layers other than the stratum corneum. In this study we applied a voltage of 50 mV and used a 3-electrode system. The 3-electrode system consisted of a measuring electrode (M), a countercurrent electrode (C), and a reference voltage electrode (R), which ensured a constant applied voltage across the stratum corneum beneath the M electrode. The placement of the electrodes on enrolled children was on the palm of the hand according to the Fig. 2.

The distance between each electrode has been at least 7 mm as recommended by the manufactor. The electrode named M was placed at the hypothenar emminence because this area on the palm gives highest stability and thus less movement artifacts; the electrode named R was placed below the third finger and the electrode named C was placed on the hypertenar side of the hand.

**Figure 3:**
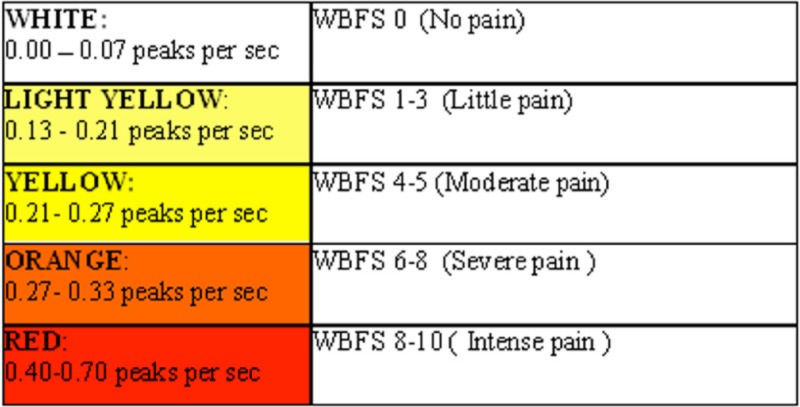
Conversion table provided by MedStorm.

We used the Med-Storm software version 1.0.0.33 (Med-Storm Innovation AS, Oslo, Norway). The Med-Storm manual contains an index where the SC fluctuations per sec are transformed to a graded score from zero to ten (Fig. 3) [available at www.med-storm.com/] . This transformation is empirical and based, among other things, on pain referred by adults (Ledowski et al., 2007); similar results have been reported for children: sensivity in 5–7 years old children is 97.0% while specificity is 72.9%; sensivity in 8–16 years old subjects is 85.2% while specificity is 67.1% (Hullett et al., 2009).

### Pain evaluation procedure with the WBFS and the skin conductance test

The WBFS was applied in the first minute after venipuncture; at that time, the children were asked to indicate on the picture (see Fig. 1) the level of pain they felt throughout the entire procedure. In the case of the SCT, electrodes were distributed over the palm of the hand. We divided the process of the measurement into three steps: preparation of the child; insertion of the needle; extraction of the needle. Skin conduction fluctuations per second were recorded at the same anatomic location, namely the antecubital fossa (see Fig. 2), and by the same operator (VM) using the BD Vacutainer 367286 blood collection set (21 G × 3/4^″^ × 12^″^ [0.8 × 19 mm × 305 mm]; Becton Dickinson & Company, Plymouth, UK). SCF was measured in each subject during venipuncture at least 10 min. They were recorded for 1 min before preparation of the child, during insertion of the needle, and for 1 min after extraction of the needle (Storm, 2008). Children and parents provided consent to the procedure, and nurses carried out the distraction techniques routinely used in our children’s hospital.

## Statistical analysis

The sample size was calculated to find a relationship in the evaluation of pain using the two methods, with an estimated correlation coefficient (based on a pilot study) of 0.175, α = 0.05 and β = 0.55, 143 patients were needed per group.

We tested the normality of the two variables (WBFS and SCF) using the Kolmogorov-Smirnov test. Because of the rejection of normality assumption, we report data as median and interquartile range and we performed non parametric tests. We used the Wilcoxon signed rank test and the Spearman correlation coefficient to compare the scores obtained with WBFS and SCF. We also compared the scores of children with or without previous exposure to venipuncture and children at different ages using the Wilcoxon sum rank test. To determine the relevance of the studied variables in relation to pain, we performed a multivariate linear regression model. Statistical analyses were performed by using SAS 9.2 (SAS Institute Inc., Cary, NC, USA).

## Results

Of the 195 patients assessed for eligibility, 150 children (78 girls and 72 boys) aged from 5 to 16 years were included in the study: 23 children did not meet the inclusion criteria, and 22 declined to participate in the study). One hundred children were undergoing venipuncture for routine hematologic tests for the first time (“never exposed”), while 50, who were affected by a chronic disease, had previously undergone venipuncture (see Table 1).

**Table 1:** Characteristics of the 150 children enrolled in the study.

Age	Mean years	SD	Median year	Interquartile range
				(25th–75th)
Mean age (year)	10.49	0.36	11.00	7–13
Gender		Boys		Girls
		72 (48%)		78 (52%)
Exposure to venipuncture				
Never exposed			100 (66.7%)	
Previously exposed			50 (33.3 %) of which diagnosis:	
				21 (42%) Endocrine diseases
				18 (36%) Gastrointestinal disorders
				11 (22%) Diabetes mellitus type 1

Table 2 shows the number of SCF 1 min before venipuncture, and during insertion and removal of the needle. Mean duration of procedures was 5.5 min (DS ± 1.2). The number of SCF per sec was significantly higher during venipuncture (0.33 fluctuations/s) than before venipuncture and during removal of the needle (*p* < 0.0001).

**Table 2:** Skin conductance values in relation to health status before venipuncture, during insertion of the needle and during removal of the needle.

	Before venipuncture	Insertion of needle	Removal of needle	*P*
All children (150)	0.27 (0.20–0.33)	0.33 (0.27–0.40)	0.20 (0.13–0.27)	<0.0001
Never exposed (100)	0.20 (0.13–0.27)	0.40 (0.33–0.53)	0.17 (0.07–0.27)	<0.0001
Exposed (50)	0.27 (0.20–0.33)	0.33 (0.27–0.40)	0.22 (0.17–0.27)	<0.0001

**Notes.**

Unit of measurement: Peak per second of Siemens

Table 3 shows the WBFS and SCF results. The self-report pain score was significantly lower than the SCF score (median WBFS score: 2; median SCF score: 5, *p* < 0.0001). This difference was consistent among all subgroups except for the younger children previously exposed to venipuncture, in whom the results of WBFS were consistent with the results of SCF (median score of 6 versus 6, *p* 0.27). Skin conductance fluctuations per sec were similar across all subgroups. Differently, in children below the age of 8 years, pain evaluated with the WBFS was less intense in those never exposed to venipuncture versus those with previous exposure (median of 2 versus 6, *p* 0.0001). Correlation analyses confirmed these results: WBFS scores are lowly correlated with SCF scores in older children and in children without previous experience of venipuncture (Table 4).

**Table 3:** Wong–Baker Faces Scale score and skin conductance per sec scores according to previous exposure to venipuncture and age.

	Wong–Baker scale	Estimated pain scores (0–10) based on SC fluctuation	*p*-value
	median (range)	median (range)	
All children (150)	2 (0–4)	5 (4–6)	<0.0001
Status			
Never exposed to venipuncture (100)	2 (0–4)	5 (4–6)	<0.0001
Previously exposed to venipuncture (50)	2 (2–6)	5 (4–6)	0.04
*p-values*	0.004	0.86	
Age			
<8 years old (52)	4 (2–5)	6 (4–6)	0.002
8 + years old (98)	2 (0-4)	5 (4–6)	<0.0001
*p-values*	0.0001	0.30	
Sex			
Male (72)	2 (1–4)	6 (4–6)	<0.0001
Female (78)	2 (0–4)	5 (4–6)	<0.0001
*p-values*	0.19	0.13	
<8 years old			
Never exposed to venipuncture (37)	2 (2–4)	6 (4–6)	<0.0001
Previously exposed to venipuncture (15)	6 (4–10)	6 (4–8)	0.27
*p-values*	0.0001	0.89	
8 + years old			
Never exposed to venipuncture (63)	2 (0–4)	5 (4–6)	<0.0001
Previously exposed to venipuncture (35)	2 (0–4)	5 (4–6)	0.0005
*p-values*	0.14	0.83	

**Notes.**

Method of comparison used: Wilcoxon test (*p* < 0.0001).

**Table 4:** Spearman correlation coefficient (*p*-value) between WBFS score and SC per sec score.

	Spearman coefficient (*p*-value)
All subjects	0.30 (0.0002)
No previous experience of venipuncture	0.28 (0.05)
Previous experience of venipuncture	0.35 (0.0004)
Children <8 years old	0.35 (0.0004)
Children >8 years old	0.20 (0.15)

The results of the linear regression model (Table 5) show that age and previous exposure are important determinants of WBFS. In fact, the score was 1.81 points lower in older than in young children, and 0.86 points lower in children never exposed than in exposed children (both statistically significant, *p* < 0.0001). The *r*^2^ of the model is 0.23, which shows that age and health status are involved in WBFS pain assessment by children. On the contrary, neither age nor health status affected the SCF score (the *r*^2^ approaches zero). Sex does not seem to affect either scale.

**Table 5:** Multivariate linear regression for the Wong–Baker Faces Scale scores and skin conductance fluctuations per sec scores.

	β	*p*-value	*r*^2^ of the model
Dependent variable = Wong–Baker faces scale			
Age	−1.81	<0.0001	
Previous experience of venipuncture	−0.86	<0.0001	
Sex	0.65	0.07	0.23
Dependent variable = Skin conductance			
Age	−0.42	0.26	
Previous experience of venipuncture	−0.09	0.65	
Sex	−0.39	0.27	0.00001

## Discussion

Painful invasive procedures such as venipuncture are usually inevitable during the routine care of children whether healthy or sick. Pediatric pain experiences are a consequence of an intricate interplay of genetic, experiential, and developmental factors (Walco, 2008). Therefore, pain assessment should not be an isolated element, but an ongoing and integral part of total pain management particularly in children (Taddio et al., 2011). Face scales are frequently used as self-report measures of pain intensity in research and clinical practice, and the Royal College of Nursing has identified the WBFS as suitable for peri-procedural pain (Roeggen, 2009; Tomlinson et al., 2010). Of the various objective pain assessment tools, one of the most widely investigated procedures is SCF per sec. In this study, we assessed the pain perceived by children during venipuncture using the two tools simultaneously: the WBFS (as subjective scale) and SCF per sec (as objective scale). The same stimulus, insertion of a needle, was used for children of different ages with and without previous exposure to venipuncture. Since the stimulus was the same for all children enrolled in the study, it was expected that the responses would be similar for the two methods and across all patient groups. To our knowledge, this is the first study to evaluate the WBFS and the SCF test simultaneously, and the first to use SCF to measure pain during venipuncture.

We found that SCF per sec were significantly higher during venipuncture than before the procedure. Therefore, the increase in the number of SCF per sec can be interpreted as a response to a painful procedure related to the insertion of the needle, whereas the SCF per sec recorded before beginning insertion of the needle are related to emotional stress and fear perceived by children (Pereira-da-Silva et al., 2012). In addition, we found that the increase in SCF per sec was not influenced by age or previous experience of venipuncture. Similarly, in previous studies conducted in infants, health status, gestational age and postnatal age did not affect SCF per sec during painful procedures or when the infant was calm (Munsters et al., 2012; Pereira-da-Silva et al., 2012; Roeggen, Storm & Harrison, 2011; Storm et al., 2002). Furthermore, using skin conductance monitoring, Røeggen et al. reported a very low variation between and within infants on the same discomfort level (behavioral state 1) (Roeggen, Storm & Harrison, 2011).

On the contrary, we found that the WBFS was affected by both age and previous experience of venipuncture although the correlation between the two methods is only low. In fact, the WBFS results differed between children below the age of 8 years with experience of venipuncture and older, venipuncture-naive children. This seems to be in line with the report by von Baeyer et al. that previous painful events play a role in the anticipation and evaluation of future pain experience (von Baeyer et al., 2004). In the older, never-exposed children, the lower values of WBFS with respect to SCF probably reflect an underestimation of pain related to a lack of sensitization, together with good practice of the team nurse and a comfortable environment during the procedure (von Baeyer et al., 2004). However, we cannot exclude that the distraction techniques used during application of the electrodes in the SCF test could have affected the children’s pain scores.

Pagé et al. used various pain scales to assess acute postoperative pain in children and observed that children aged 8–18 years find the faces scales the easiest to use (Pagé et al., 2012). Moreover, in a comparative prospective evaluation of responsiveness to self-report scales, Connelly et al. reported that patients tended to rate pain intensity higher with numerical rating scale than with a visual analog scale (Connelly & Neville, 2010).

A recent systematic review of cross-cultural comparison studies of child, parent, and health professional outcomes associated with pediatric medical procedures revealed that cultural factors may be associated with children’s pain experiences when elicited by medical procedural pain, specifically children’s pain behavior, but the authors noted that research using more sophisticated research methods is needed to develop culturally sensitive behavioral pain measures (Kristjansdottir et al., 2012). In this context, Drendel et al. reported that a variety of factors could affect the self-report of pain in children, and that optimizing the use of pain assessment remains a challenge for the health care provider in the emergency setting of pediatric practice (Drendel, Kelly & Ali, 2011).

Our findings using skin conductance monitoring are in line with those reported by Hullet et al. (Hullett et al., 2009) who monitored postoperative pain using a postoperative pain score in children. In fact, they found that the best accordance between a self-reported pain score and SCF per sec occurred in children between the ages of 4 and 8 years. Interestingly, two studies of postoperative pain in children showed that anxiety did not affect the skin conductance analysis (Choo et al., 2010; Hullett et al., 2009) and in one of the studies there was no correlation between skin conductance activity and WBFS scores (Choo et al., 2010), unlike our findings. The lack of correlation between the two procedures may be due to the low specificity of skin conductance to identify moderate pain or it could indicate that WBFS pain scores are influenced by age and health status as shown in our study.

A potential limitation of this study is the lack of previous scientific literature about the comparison between SCF and WBFS and this issue makes more difficult to establish the study design. Another limitation is the difficulty to convert SCF data in a numerical scale that could be compared with WBFS scores. Additionally there is no universally accepted lower age limit for the self-reporting of pain in the use of WBFS (Hicks et al., 2001). Further, fear and anxiety may bias pain reporting and interfere with attempts at measuring pain intensity; although the WBFS are reported to have content validity (Garra et al., 2010).

Lastly our sample size calculation is probably not adequate to examine the sub group analysis (previously exposed to venipuncture versus not).

In conclusion, this simultaneous evaluation of the WBFS and SCF in the assessment of pain in children undergoing a routine medical procedure showed that, although both tools can be useful for research and in clinical practice, SCF produced uniform data, whereas the WBFS was affected by age and previous venipuncture experience thereby providing new data about school-age children. If verified in other studies, our results should be taken into account when using these tools to evaluate pain in children and for providing more adequate pain care routine. Additional researches on positioning and of nonpharmacologic and pharmacologic interventions are needed, including larger samples sizes and expanded ages.

**Abbreviations**
SCF: Skin conductance fluctuations
WBFS: Wong–Baker faces pain rating scale
